# Gefitinib (‘Iressa’, ZD1839) inhibits the growth response of bladder tumour cell lines to epidermal growth factor and induces TIMP2

**DOI:** 10.1038/sj.bjc.6601768

**Published:** 2004-04-06

**Authors:** J E Nutt, H P Lazarowicz, J K Mellon, J Lunec

**Affiliations:** 1 Northern Institute for Cancer Research, Medical School, Framlington Place, Newcastle upon Tyne NE2 4HH, UK; 2 Clinical Sciences Unit, Leicester General Hospital, Leicester LE5 4PW, UK

**Keywords:** gefitinib, epidermal growth factor receptor, bladder cell lines, MMPs, TIMPs

## Abstract

The effect of EGF stimulation and its inhibition with gefitinib (‘Iressa’, ZD1839), an epidermal growth factor receptor (EGFR) tyrosine kinase inhibitor, has been investigated in two EGFR-positive human bladder tumour cell lines, RT112 and RT4. The growth of RT112 cells in a medium containing 10% foetal bovine serum was inhibited by 50% with 10 *μ*M gefitinib, whereas this dose completely inhibited RT4 cell growth. Cells were more sensitive to growth inhibition in the serum-free medium. Increased growth of cells in the serum-free medium was observed with 10 or 50 ng ml^−1^ EGF and the proliferative effect of EGF stimulation in both cell lines was inhibited in the presence of 1 *μ*M, but not 0.1 *μ*M gefitinib. Zymography of the conditioned medium from RT112 cells treated with EGF and gefitinib showed a decrease in matrix metalloproteinase 2 (MMP2) concentrations. Western blot analysis showed that tissue inhibitor of metalloproteinase 1(TIMP1) increased in the conditioned medium from RT112 cells treated with EGF, and this was partially inhibited with both 1 and 5 *μ*M gefitinib. Conversely, TIMP2 decreased with EGF stimulation and this was reversed with gefitinib. Tissue inhibitor of metalloproteinase 1 had no effect on the growth of either cell line. These studies show alterations in the balance of MMPs and their inhibitors in EGF-stimulated bladder tumour cells, which are reversed by gefitinib, suggesting gefitinib should be investigated for its effect on human bladder tumours.

Growth factors and their receptors are important in tumour development and progression. Several studies have demonstrated that the presence of epidermal growth factor receptor (EGFR) in bladder cancer is associated with high tumour stage and grade, and is a strong independent predictor of tumour progression and poor long-term survival (Lipponen and Eskelinen, 1994; Mellon *et al*, 1995). Bladder cancer is the fifth most common cancer in men, and transitional cell carcinoma of the bladder can be broadly divided into superficial (Ta and T1) or muscle invasive (T2, T3 and T4) forms. Approximately 50–70% of superficial tumours recur and 10–20% of these will become invasive. At present there is no reliable method to predict which superficial tumours will show invasive progression or metastasise.

Epidermal growth factor receptor is a member of the erbB family of cell surface receptors, which comprises four homologous receptors: EGFR (erbB-1/HER1); erbB-2 (HER2/neu); erbB-3 (HER3) and erbB-4 (HER4). These receptors are composed of an extracellular ligand-binding domain, a transmembrane domain and an intracellular protein kinase domain. Ligand-binding activates the EGFR by inducing homo- or hetero-dimerisation with other members of the erbB family, resulting in autophosphorylation of both intracellular tyrosine kinase domains. This initiates a cascade of intracellular signalling events (Olayioye *et al*, 2000).

Epidermal growth factor receptor signalling is critical not only for cell proliferation but also in other processes crucial to cancer progression, and EGFR is therefore a target for anticancer therapies. One approach for the therapeutic blockade of EGFR signalling has been the discovery and development of low molecular weight compounds that inhibit the activation of the EGFR tyrosine kinase. Gefitinib (‘Iressa’, ZD1839) is an orally active EGFR tyrosine kinase inhibitor (EGFR-TKI). It is a low molecular weight, synthetic quinazoline derivative that blocks signal transduction pathways implicated in the proliferation and survival of cancer cells (Wakeling *et al*, 1996). Gefitinib is currently undergoing clinical cancer trials, both as a single agent or in combination with established cytotoxic agents (Ciardiello and Tortora, 2001). This, and other EGFR-TKIs, has recently been reviewed (Wakeling, 2002).

The matrix metalloproteinases (MMPs) are a family of proteolytic enzymes present in both normal and pathological tissues in which matrix remodelling is involved, including embryonic development, wound healing, arthritis, angiogenesis and tumour invasion and metastasis. The MMPs, which are secreted or membrane bound, contain a zinc atom at their active site. The expression of MMPs is primarily regulated at the level of transcription, and MMPs are synthesised as latent proenzymes requiring extracellular proteolytic activation. Activity is also regulated by endogenous tissue inhibitors of metalloproteinases (TIMPs), which bind to activated MMPs in a 1 : 1 molar stoichiometry. In some cell lines, TIMPs are reported to have a stimulatory effect on cell growth. Both the MMPs and TIMPs have been the subject of several recent reviews (Cox and O'Byrne, 2001; Egeblad and Werb, 2002; Vihinen and Kahari, 2002).

In bladder cancer patients, urinary MMP1 concentration has been reported to increase significantly with increased stage and grade of tumour (Nutt *et al*, 1998). Matrix metalloproteinase 2 and MMP9 activity was significantly enhanced in urine from patients with highly invasive tumours (Sier *et al*, 2000), and also found to be associated with high stage and grade of bladder tumours (Gerhards *et al*, 2001). Patients with muscle invasive tumours were found to have higher urinary concentrations of TIMP1 than those with superficial tumours (Durkan *et al*, 2001).

Since EGFR in bladder cancer is associated with high stage and grade of tumour, and urinary concentrations of EGF are high, this study has investigated the effect of gefitinib on EGF-stimulated growth in two human bladder tumour cell lines, and the effect on MMP2, TIMP1 and TIMP2 levels found in the conditioned medium from the cultured cells. Owing to its previous reported effect on cell growth, the specific effect of TIMP1 on the growth of bladder cell lines was also investigated.

## MATERIALS AND METHODS

### Cell culture

Two human bladder tumour cell lines, RT4 and RT112, were routinely grown in the RPMI medium containing 10% foetal bovine serum (FBS). These cell lines differ in their EGFR numbers, with RT4 and RT112 cells containing 1.9 × 10^4^ and 9 × 10^3^ receptors per cell, respectively, as measured by FACS analysis (Brotherick *et al*, 1994). All cells were negative for mycoplasma. Growth of cells was measured using the sulphorhodamine B (SRB) assay (Skehan *et al*, 1990). Cells were seeded in 96-well plates at 4 × 10^3^ or 1 × 10^4^ cells per well and incubated for 24 h. The medium was removed and the cells washed with phosphate-buffered saline (PBS) and fresh medium either with or without FBS was added for a further 24 h. In a subset of experiments, serum-free medium was used to remove exogenous growth factors from cells to be treated with EGF. The medium was then replaced with the test medium containing EGF (human recombinant, Sigma-Aldrich, Poole, UK) at 10 or 50 ng ml^−1^ or gefitinib (from 0.1–10 *μ*M) alone or in combination with EGF for the required time. The cells were fixed with 25 *μ*l 50% trichloroacetic acid, stained with SRB and the plates were scanned for absorbance at 570 nm.

For the conditioned medium used in zymograms and Western blotting, cell culture was as previously described using 145 cm^2^ tissue culture dishes and cells in the serum-free medium containing 0.1% bovine serum albumin (Nutt *et al*, 1998). The test medium contained EGF (10, 50 or 100 ng ml^−1^) or gefitinib (1 or 5 *μ*M) or a combination, and cells were incubated for a further 48 h. The medium was removed, centrifuged to remove any cells and stored at −20°C prior to 10-fold concentration using Centricon-10 concentrators (Millipore (UK) Ltd, UK).

Cell lysates were used in Western blotting analysis to verify the relative levels of EGFR. Phosphorylation of EGFR of cells grown in the serum-free and serum-rich medium at basal levels and following 10 min stimulation with 10 ng ml^−1^ EGF were also determined by Western blotting, using the Phospho-EGF Receptor (Tyr992) polyclonal antibody (Cell Signalling Technology, USA).

The effect of TIMP1 on cell growth in both RT112 and RT4 cell lines was studied using the SRB assay as above. A stock solution of human TIMP1 (Chemicon International Ltd) was prepared in PBS at 10 *μ*g ml^−1^ and added to cells in the serum-free medium for up to 4 days, in a range of final concentrations of 1–500 ng ml^−1^. Cells were observed daily with the microscope before fixation.

### Zymography

Zymography was performed using 10% polyacrylamide gels with gelatin incorporated as a substrate for proteases (Zymogram Ready Gels, Biorad, UK). Samples were mixed with a 5 × sample buffer (Herron *et al*, 1986) adapted as follows: 20 ml sample buffer was prepared using 12.5 ml 0.5 M Tris-HCl buffer pH 6.8, 2.5 g SDS, 1 g sucrose and 0.02 g bromophenol blue. Stocks were stored frozen and aliquots thawed as required. A 15 *μ*l total sample, generally 12 *μ*l sample with 3 *μ*l sample buffer, was loaded onto the gel. Electrophoresis was performed at room temperature in Tris/glycine/SDS buffer for 1.5 h at 100 V and the gels were renatured in 2.5% Triton X-100 for 30 min at room temperature to remove SDS from the gels and reactivate any MMPs present. The gels were incubated overnight at 37°C in a shaking water bath in development buffer (50 mM Tris-HCl pH 7.5, 200 mM NaCl, 5 mM CaCl_2_, 0.02% Brij-35). They were then stained in 0.5% Coomassie Blue R-250 in 40% methanol, 10% acetic acid for 1.5 h and destained in 40% methanol, 10% acetic acid for 1.5 h with a rinse and two changes of destain solution to visualise digested bands in the gelatin matrix. The gels were photographed using a digital camera and quantification was performed using Molecular Dynamics ImageQuant software.

### Western blot analysis

Western blotting was performed to investigate the presence of TIMP1 and TIMP2 in the conditioned media. Following SDS–PAGE electrophoresis and electroblotting (Towbin *et al*, 1979), membranes were blocked using a 5% milk solution prior to incubation with specific antibodies. Antibodies were obtained from Oncogene Research Products and were used at a concentration of 1 *μ*g ml^−1^. Tissue inhibitor of metalloproteinase 1 (Ab1) and TIMP2 (Ab1) and a secondary HRP-conjugated antibody (Goat-antimouse, Dako, Ely, UK) were used and detection was performed using enhanced chemiluminescence (Amersham Biosciences, UK).

## RESULTS

### EGF receptors in cell lines

**Figure 1:**
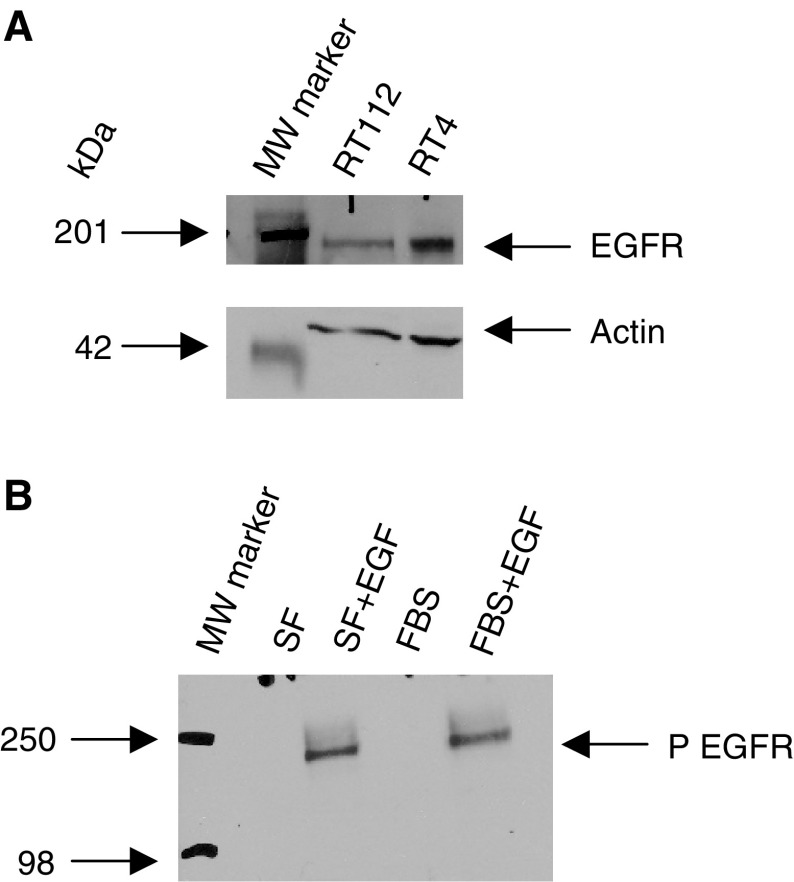
(**A**) Western blot analysis of cell lysates from RT112 and RT4 cells showing the relative levels of EGFR. Actin is shown to verify equal loading. (**B**) Western blot analysis of cell lysates from RT112 cells grown in the serum-free (SF) or serum-rich (FBS) medium without or with 10 min treatment 10 ng ml^−1^ EGF. Phosphorylated EGFR (P EGFR) was detected with a Tyr992 antibody.

The results of Western blotting analysis of the RT4 and RT112 cells to confirm the relative levels of EGFR are shown in Figure 1(A)

. More receptors are present in the RT4 cells, confirming previous results of FACS analysis.

No phosphorylated EGF receptors were detectable in cells grown in either the serum-free or serum-rich medium without additional stimulus. Phosphorylated EGFR was apparent following 10 min stimulation with EGF in both the media. Results in Figure 1(B) show the use of phospho-EGF receptor (Tyr992) antibody in RT112 cells. Similar results were obtained with Tyr845, 1045 and 1068 antibodies.

### Effect of gefitinib on cell growth

**Figure 2:**
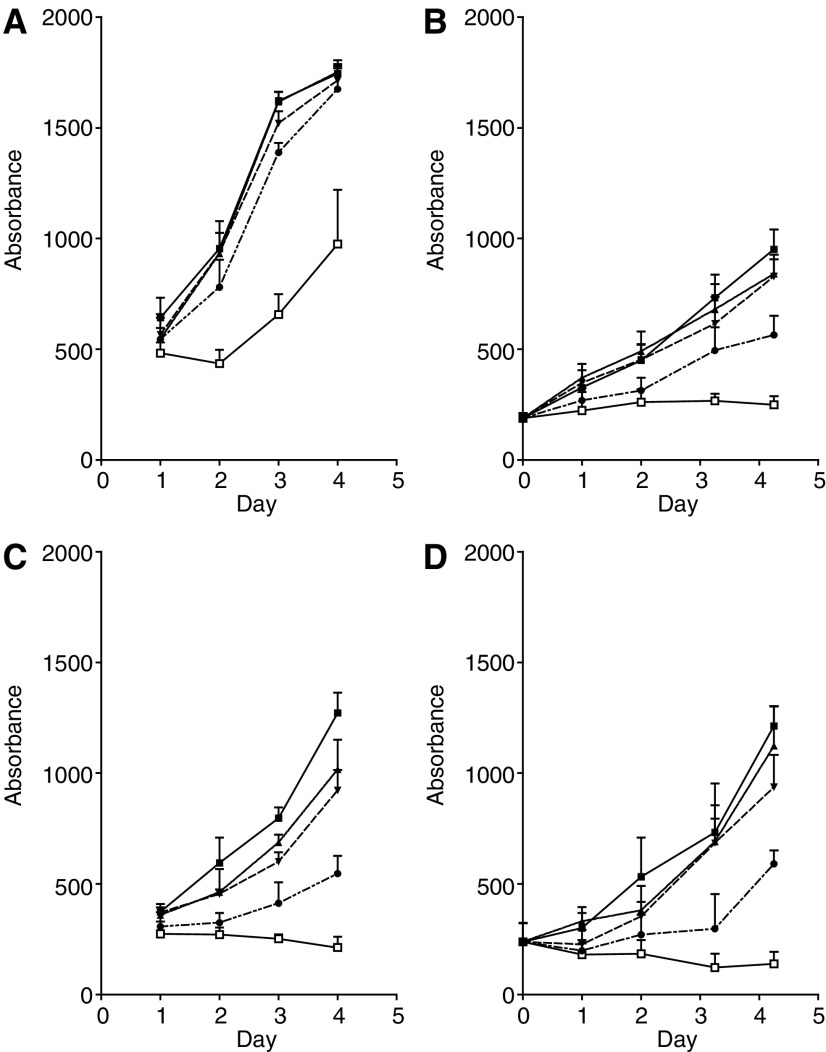
Cell growth, measured by the SRB assay, of RT112 cells (**A** and **B**) and RT4 cells (**C** and **D**) in the medium containing 10% FBS (**A** and **C**) or in the serum-free medium (**B** and **D**) with increasing concentrations of gefitinib. Error bars show ±s.d. of six replicates in one representative experiment. ▪ Control medium; ▴ 0.1 *μ*M gefitinib; ▾ 1 *μ*M gefitinib; • 5 *μ*M gefitinib; □ 10 *μ*M gefitinib.

Human bladder cancer cell lines RT112 and RT4 were exposed to continuous treatment with gefitinib at concentrations 0.1–10 *μ*Min both the serum-rich (10%) and serum-free medium. The results are shown in Figure 2

. In the serum-rich medium, gefitinib from 0.1–5 *μ*M had no effect on the cell growth of RT112 cells over 4 days, whereas 10 *μ*M gefitinib reduced the growth by 50% (Figure 2A). In contrast, the RT4 cells grown in the serum-rich medium were more sensitive to gefitinib, with 5 *μ*M giving 50% reduction in growth, and 10 *μ*M not only inhibiting cell growth but also showing evidence of a reduction in cell number compared with initial cell seeding levels (Figure 2C). Similar results were obtained for RT4 cells grown in the serum-free medium (Figure 2D). However, in the serum-free medium, RT112 cells were markedly more sensitive to gefitinib, with 5 *μ*M decreasing cell growth by 50% and 10 *μ*M inhibiting cell growth (Figure 2B).

### Effect of gefitinib on EGF-stimulated cell growth

**Figure 3:**
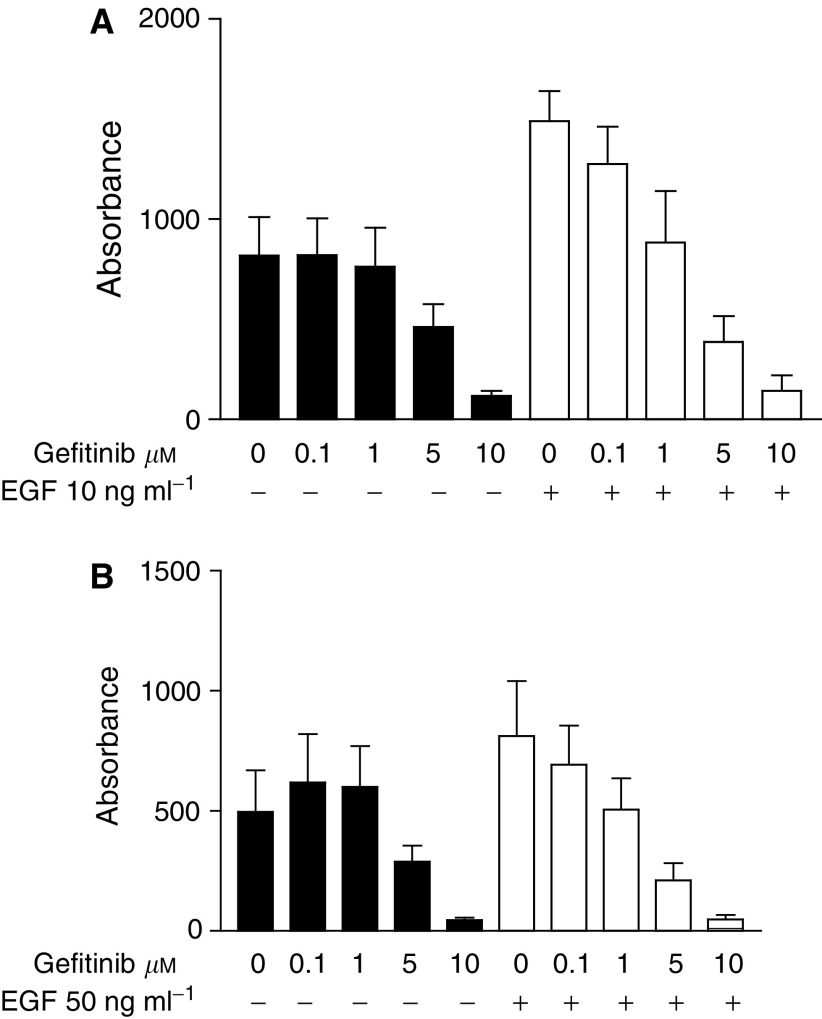
Effect of EGF and gefitinib on cell growth of RT112 (**A**) and RT4 (**B**) cells in the serum-free medium after 4 days in culture. Error bars show ±s.d. of six replicates in one representative experiment. Filled bars show results with gefitinib alone, plain bars in the presence of EGF, at the concentrations indicated.

The results of EGF growth stimulation in the serum-free medium and the inhibition of EGF-stimulated cell growth with gefitinib are shown in Figure 3

. In the RT112 cell line, following treatment with gefitinib and 10 ng ml^−1^ EGF for 4 days, EGF-stimulated growth was not inhibited by 0.1 *μ*M gefitinib, but was inhibited at 1, 5 and 10 *μ*M gefitinib (Figure 3A). At these higher concentrations, comparable growth curves were obtained with gefitinib alone or in the presence of EGF. Similar results were obtained using 50 ng ml^−1^ EGF, with 1 *μ*M gefitinib inhibiting the EGF stimulatory effect (data not shown).

The results from the RT4 cell line following treatment with 50 ng ml^−1^ EGF and 0.1–10 *μ*M gefitinib for 4 days are shown in Figure 3B. Again, comparable results were seen with the two doses of EGF (results not shown), and only 0.1 *μ*M gefitinib did not inhibit the EGF-stimulated growth.

### Effect of gefitinib and EGF on MMP secretion

**Figure 4:**
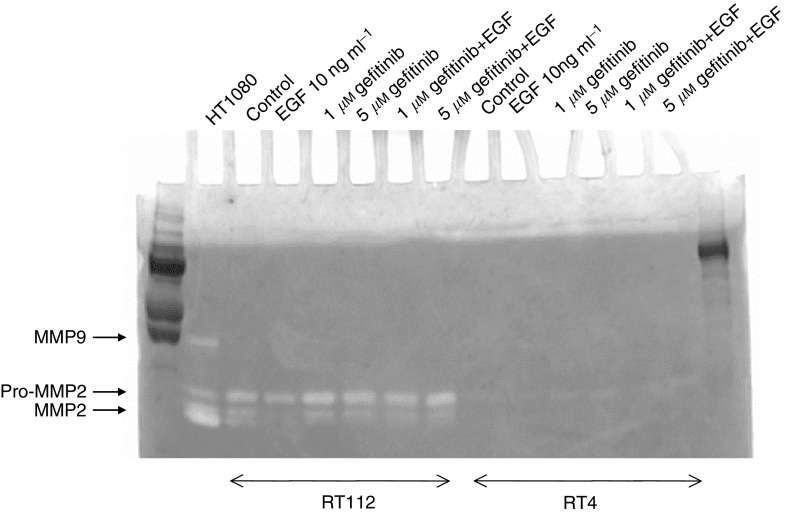
Zymogram of the serum-free conditioned medium from RT112 and RT4 cells treated with EGF and gefitinib for 48 h. The conditioned medium from HT1080 cells treated with phorbol ester is included as a control to show bands of MMP9 and MMP2 activity.

The conditioned media from cells treated with 1 or 5 *μ*M gefitinib alone or with 10 ng ml^−1^ EGF were used for zymogram analysis. The result of a zymogram, representative of the conditioned media from three separate cell culture experiments, is shown in Figure 4

**Figure 5:**
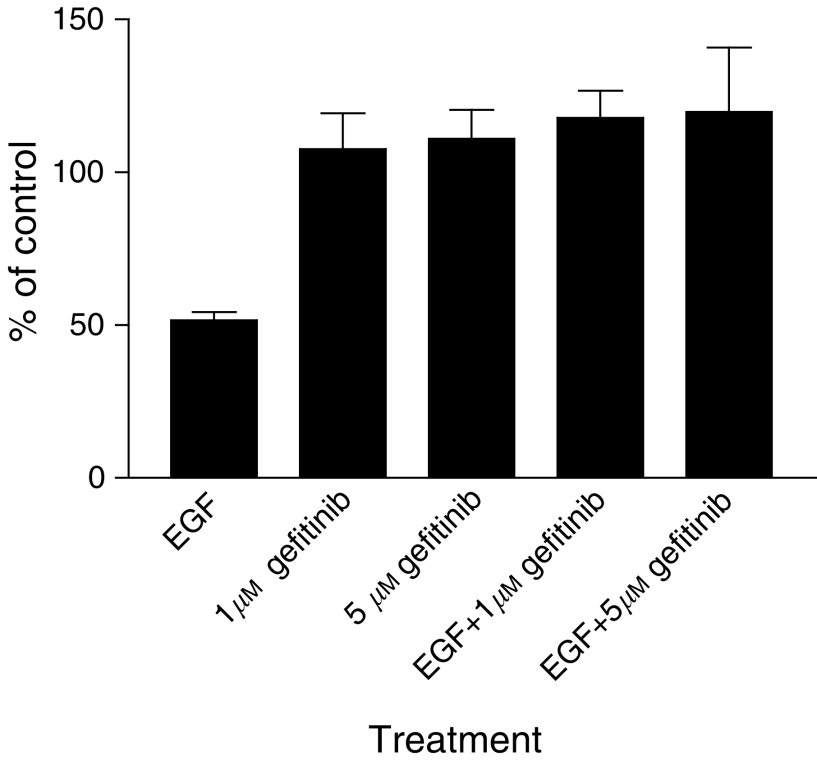
Matrix metalloproteinase 2 activity in the serum-free conditioned medium at 48 h from RT112 cells as a percentage of the 48 h control value measured by densitometry of zymograms. Columns are the mean of three separate tissue culture experiments; error bars are ±s.e.

. The medium from HT1080 cells is used as a control to demonstrate bands of MMP9 and MMP2 activity. Matrix metalloproteinase 2 was detectable in all the media from RT112 cells. The active MMP2 was decreased following continuous EGF treatment alone, but there was no change from the control levels in cells treated with gefitinib alone or in combination with EGF, demonstrating the inhibition of EGF action on the secretion of MMP2 activity by gefitinib. Both concentrations of gefitinib used gave similar results, with no indication of dose dependency of gefitinib in the concentrations used, although their effects on growth differed. From densitometry of the zymograms, the total gelatinolytic activity in treated cells as a percentage of the controls for RT112 cells is shown in Figure 5

, confirming the maximum effect on EGF-stimulated MMP2 synthesis occurring with 1 *μ*M gefitinib. In the RT4 cell culture, there were very low levels of secreted MMP2 in the conditioned media and there was no detectable effect of EGF and/or gefitinib on secreted metalloproteinases (Figure 4). There was no evidence, in either cell line, of any MMP9 being secreted into the conditioned media following treatment with EGF or gefitinib.

### Western blotting analysis for TIMP1 and TIMP2

**Figure 6:**
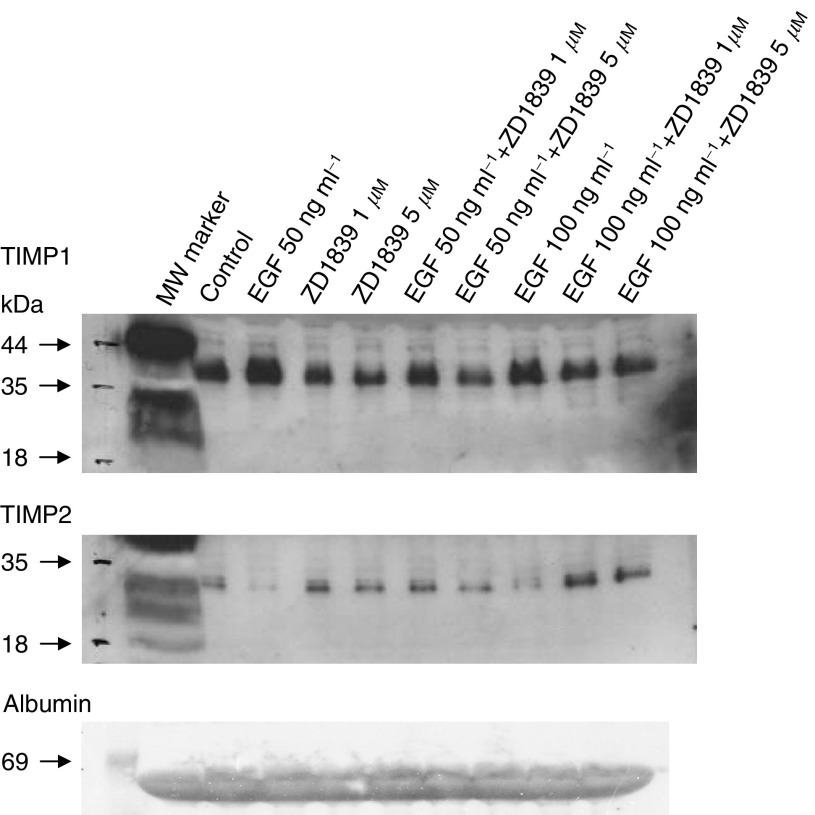
Western blot analysis for TIMP1 and TIMP2 in the conditioned medium from RT112 cells following 48 h treatment with EGF and/or gefitinib. The albumin band seen with the TIMP1 antibody verifies equal loading of samples.

The results of Western blotting analysis for the detection of TIMP1 and TIMP2 in the conditioned media from RT112 cells treated with EGF and gefitinib alone or in combination are shown in Figure 6

. Tissue inhibitor of metalloproteinase 1 levels were increased three-fold above the control levels (measured by densitometry) with both 50 and 100 ng ml^−1^ EGF. This level of TIMP1 was decreased in a dose-dependent manner when the cells were incubated with gefitinib for 48 h. This was particularly evident with 50 ng ml^−1^ of EGF. The reverse effect was observed for TIMP2, with 10% of the control level of protein being produced with 50 ng ml^−1^ EGF. The effect of EGF was abolished in the presence of both doses of gefitinib used. Equal loading of samples was seen in the albumin band.

In the conditioned medium from RT4 cells, no TIMP1 or TIMP2 was detectable in any medium by Western blotting (data not shown).

### Effect of TIMP1 on cell growth

The morphological appearance of both cell lines showed no obvious change over the 4 days treatment with TIMP1 at any of the concentrations used. There was no stimulation or inhibition of cell growth over 4 days with any concentration of TIMP1 in either cell line (data not shown).

## DISCUSSION

Strategies targeting the EGFR may well have a therapeutic potential in bladder cancer, since the receptor is frequently expressed at high levels in this disease and patients with tumours expressing high levels of EGFR tend to have a poor prognosis (Mellon *et al*, 1995). The tyrosine kinase inhibitor, gefitinib, is targeted against EGFR and is being used in clinical trials with objective responses of antitumour activity being noted in non-small-cell lung cancer, squamous cell carcinoma of the head and neck and in hormone-refractory prostate cancer (Baselga and Averbuch, 2000; de Bono and Rowinsky, 2002). The present study has investigated the effect of gefitinib on growth in two human bladder cancer cell lines and the effect on MMPs and TIMPs, both involved in tumour progression and metastasis and known to be modulated by EGF receptor activity.

The two cell lines studied showed differing sensitivities to gefitinib. In the serum-rich medium, RT4 cells were more sensitive to gefitinib than RT112 cells. This was surprising since the RT4 cells contain approximately twice the number of EGF receptors as RT112 and suggests that, in spite of the lower levels of EGF receptors, the growth of RT112 cells is more dependent on their activity. There are mixed reports on the correlation of growth inhibition by gefitinib and the EGFR status of cells. In one study, the sensitivity of a panel of human breast cancer and other epithelial tumour cell lines to gefitinib was found not to be dependent on overexpression of EGFR (Moasser *et al*, 2001), and was also suggested to be related to erbB2 expression (Anderson *et al*, 2001). However, an inverse correlation between IC_50_ and EGFR levels in a range of tumour cell lines has been reported (Magne *et al*, 2002). In another study, four different bladder tumour cell lines have been reported to show a dose-dependent inhibition of cell proliferation when treated with gefitinib, which correlated with the EGFR protein level (Meye *et al*, 2001).

In contrast, in the present study, in the serum-free medium, where there are no exogenous growth factors apart from those produced by autocrine stimulation, similar sensitivities were found, with 10 *μ*M gefitinib preventing cell growth. Gefitinib also prevented the EGF-stimulated cell growth in both RT112 and RT4 cell lines in concentrations of 1 *μ*M and above. These results are similar to a study of four EGFR-positive ovarian cancer cell lines (Sewell *et al*, 2002) where growth stimulation with TGF*α*, another ligand for EGFR, was completely inhibited by gefitinib concentrations of 0.3 *μ*M and above.

Metalloproteinases are central to both matrix degradation and remodelling, and their role in tumour invasion and metastasis is now well established. The gelatinases, MMP2 and MMP9, are particularly important in the degradation of the basement membrane. Matrix metalloproteinase 2 is reported to be constitutively expressed in a variety of cells (Benbow and Brinckerhoff, 1997), and in the present study it was found to be present in the serum-free conditioned medium of untreated cells. The level of total MMP2 (both latent and active) was, however, found to be consistently reduced in the RT112 cell line when treated with EGF, with a noticeable reduction particularly in the active form of the enzyme. This was reversed to the control levels by gefitinib alone or in the presence of EGF, consistent with the inhibition of the EGF effect on downregulation of MMP2 by the action of gefitinib on the EGFR. Matrix metalloproteinase 2 is activated by activated membrane-bound metalloproteinase MT1-MMP (Sato *et al*, 1994), and TIMP2 is also involved in the ternary complex for MMP2 activation, which releases active MMP2 from the cell surface (Strongin *et al*, 1995). Tissue inhibitor of metalloproteinase 2 also inhibits MMP2 activity directly by forming a 1 : 1 complex. The decrease in MMP2 in the conditioned medium was mirrored by the decrease in TIMP2 with EGF, which was again reversed by treatment with gefitinib. This effect of EGF is somewhat surprising since most MMPs, apart from MMP2, which lacks an AP1-binding site in its promoter region, are induced by cytokines and growth factors (Benbow and Brinckerhoff, 1997). Further studies are required to investigate whether the reduction in MMP2 protein is due to inhibition by TIMP2, increased levels of MT1-MMP on the cell surface or changes in the levels of mRNA, and hence in the levels of transcription via an alternative signalling pathway other than that involving the AP1 transcription site. Variants in the MMP2 gene have recently been reported (Price *et al*, 2001), in which an SP1-type promoter site is disrupted and displays a lower promoter activity. A study of a breast cancer cell lines stimulated with EGF showed an increase in MMP9 but not MMP2, but this increase was not inhibited by the tyrosine kinase inhibitor PD153035 (Kondapaka *et al*, 1997).

EGF and gefitinib produced opposite effects on TIMP1 and TIMP2 in the conditioned medium of RT112 cells, again with gefitinib reversing the EGF response. A previous study of urinary TIMP1 concentrations (Durkan *et al*, 2001) showed higher concentrations in muscle invasive compared with superficial tumours, and a lower MMP1 : TIMP1 ratio was found in invasive tumours. A low urinary MMP9 : TIMP1 ratio was also shown to indicate an increased risk of tumour recurrence (Durkan *et al*, 2003). Tissue inhibitor of metalloproteinase 2 and MMP2 have also been investigated in bladder cancer. Using RT–PCR, invasive bladder tumour samples were found to have higher expression of MMP2 and TIMP2 compared to superficial tumours, and high levels of MMP2, TIMP2 and MT1-MMP were associated with decreased patient survival (Kanayama *et al*, 1998). MT1-MMP has been shown to be present in both invasive and superficial bladder carcinoma cells (Kitagawa *et al*, 1998). Similar results were reported using immunohistochemical studies of TIMP2 in invasive tumours (Grignon *et al*, 1996). Serum MMP2 : TIMP2 ratios have also been studied, with higher ratios occurring in patients showing recurrence or lower disease survival (Gohji *et al*, 1996).

RT112 cells have previously been shown to invade the subepithelial capillary bed in a human bladder tumour invasion model, whereas RT4 cells were noninvasive (Booth *et al*, 1997). An increased TIMP1 secretion with EGF, seen in this study in RT112 cells (Figure 6), would initially suggest more MMP inhibition, and hence less invasion. The increase in TIMP2 in the presence of gefitinib, with or without EGF, would also indicate potentially less invasion, and would suggest a possible antimetastatic effect of gefitinib. The increase in TIMP2 would also enhance the inhibition of the increased MMP2 levels produced with EGF and gefitinib. The role of TIMPs, however, has been suggested not to be purely the inhibitory action on MMPs, but also that TIMPs have growth-promoting activity. Tissue inhibitor of metalloproteinase 2 was reported to have growth-promoting activity in a wide range of human, bovine and murine cell lines at a concentration of 10 ng ml^−1^, 10-fold lower than that of TIMP1 (Hayakawa *et al*, 1994). However, no growth-promoting activity was found at concentrations greater then 1 *μ*g ml^−1^, when matrix degradation is inhibited (De Clerck *et al*, 1994). In the RT112 and RT4 bladder cell lines studied here, no effect of TIMP1 on growth was detected, using concentrations from 1 to 500 ng ml^−1^. A recent study has shown stimulation of melanoma cell lines by TIMPs, and that during melanoma progression growth responses to TIMP1 and TIMP2 may gradually change (Hoashi *et al*, 2001).

The role of TIMPs in bladder cancer and their regulation by EGF receptor signalling pathways is obviously complex and requires further investigation. The use of gefitinib may be beneficial in bladder tumours, not only for its effect on EGFR tyrosine kinase-dependent growth but also the EGF receptor-mediated effect on TIMPs.
